# Mechanical tuning of mammalian sperm behaviour by hyperactivation, rheology and substrate adhesion: a numerical exploration

**DOI:** 10.1098/rsif.2016.0633

**Published:** 2016-11

**Authors:** Kenta Ishimoto, Eamonn A. Gaffney

**Affiliations:** 1 The Hakubi Center for Advanced Research, Kyoto University, Kyoto 606-8501, Japan; 2 Research Institute for Mathematical Sciences, Kyoto University, Kyoto 606-8502, Japan; 3 Wolfson Centre for Mathematical Biology, Mathematical Institute, University of Oxford, Oxford OX2 6GG, UK

**Keywords:** sperm motility, mammalian fertilization, hyperactivation, oviductal epithelium, sperm–zona binding

## Abstract

The great mammalian sperm race encounters numerous microenvironments to which sperm must adapt and a fundamental sperm response is the change in its waveform owing to both fluid rheology and capacitation, with the latter associated with a hyperactivated beat pattern. Hence, in this modelling study, we examine the effect of different flagellar waveforms for sperm behaviour near adhesive substrates, which are representative of epithelia in female tract sperm reservoirs and the zona pellucida (ZP), which surrounds the mammalian egg. On contact with an adhesive surface, virtual sperm rotate to become nearly tangential with the surface, as generally observed. Hyperactivation also induces many effects: sperm exert greater forces on the substrate and periodically tug way from adhesions under circumstances reflecting binding at sperm reservoirs, but with extensive fluid elasticity, as found in the cumulus surrounding the ZP, sperm instead continually push into the substrate. Furthermore, with weak adhesion, rheological media increase the duration hyperactivated sperm remain in the proximity of a substrate. More generally, such results predict that changes owing to both hyperactivation of the flagellar waveform and the rheology of the surrounding medium provide a means of tuning sperm behaviour near, or attached to, adhesive substrates.

## 1. Introduction

Hundreds of millions of sperm compete in the race to the egg within the mammalian female reproductive tract, where there are numerous microenvironments to which successful sperm must adapt, such as confined geometries, rheological media, immunological responses and varying pH levels [1]. However, unlike most cells, sperm do not have recall to gene expression, and thus novel proteins in response to different mechanical and biochemical cues. A fundamental sperm response within the fallopian tubes during its journey is simply the alteration of the flagellar waveform with the induction of capacitation, which, together with numerous physiological changes [2], induces hyperactivation, namely a large amplitude, slow frequency and asymmetric flagellar beat [3–5]. However, the details of the flagellar waveform and sperm behaviour are also highly contingent on the rheology of fluid within the female tract, which provides another means of regulating sperm behaviour, as illustrated by the rheology of cervical mucus [1,6], which dictates the prospect of sperm progression through the cervix during the menstrual cycle.

**Figure 1.**
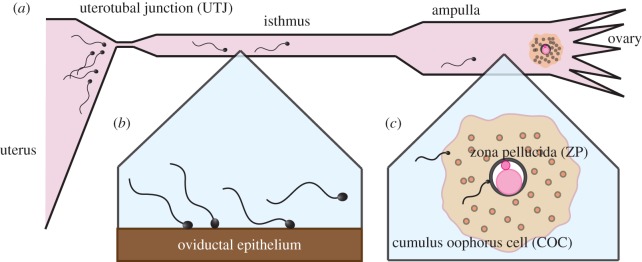
(*a*) A schematic of mammalian sperm motility in the female oviduct. The progression of spermatozoa to the oviduct is hindered by the narrow channel from the uterus, known as the uterotubal junction (UTJ). (*b*) Sperm detachment in the oviductal epithelium at the isthmus for mammals that exhibit oestrous. Sperm bind to female tract epithelium and, as ovulation approaches, hyperactivation accompanies sperm detachment for further progression towards the egg. (*c*) A schematic of the sperm–cumulus and sperm–ZP interactions during an egg–sperm encounter in the ampulla.

It is well known that capacitation and hyperactivation are necessary for successful fertilization, and have multiple functional roles in the female reproductive tract. For instance, in oestrous (heat-exhibiting) mammals, once sperm have entered the oviducts via the narrow channel of the uterotubal junction (figure 1*a*), they can bind with the oviductal isthmic epithelium (figure 1*b*), thus creating reservoirs of sperm. As ovulation approaches, these reservoir sperm become hyperactivated, and the resulting flagellar changes are considered to enhance detachment from the epithelium for further progression towards the egg [7,8] and hyperactivation is also recognized as enhancing the penetration of rheological media and for navigating around mucus pockets during this onward journey [1].

In particular, on approaching the egg, sperm have to traverse its surrounding vestments, and the first that they encounter is the cumulus oophorus cell (COC) matrix or simply *cumulus* (figure 1*c*), which consists mainly of hyaluronic acid [1,2] and is both very viscous and elastic [9]. While hyperactivated sperm do not progress well in watery-like media, their flagellar waveforms change extensively and they progress more readily in highly viscous media compared with their non-hyperactivated counterparts [10], and observations also indicate that sperm progression into the cumulus is improved by hyperactivation [11]. On passing through the cumulus, the sperm eventually encounters the *zona pellucida* (ZP), a tough glycoprotein layer, which it must penetrate (figure 1*c*). The importance of the hyperactivated waveform for the sperm–ZP interaction has been known since the early 1980s [12], and the enhancement of ZP penetration owing to hyperactivation has been experimentally observed [13]. Furthermore, sperm binding and rheology at the ZP has a fundamental impact in that, first, binding is necessary for sperm penetration in the absence of cumulus, as evidenced by gene-disruption experiments of aberrant zona binding mice spermatozoa [2] and also that fertilization can still proceed for such spermatozoa in the presence of cumulus [14–16].

There has also been extensive mathematical and computational modelling associated with these aspects of the great sperm race [17–23]. In particular, sperm detachment from oviductal isthmic epithelia has been recently explored, with Curtis *et al.* [20] using resistive force theory (RFT) [24] to confirm the mechanical consistency of hyperactivation contributing to epithelial detachment. Simons *et al.* [21] drew the same conclusion on extending this study using the slender body approximation, which has a greatly improved accuracy, with the additional inclusion of a spring bond model for substrate adhesion, though still with the approximations that sperm motion is confined in two-dimensional space, and the sperm head is simplified to a point-like object. There have also been extensive RFT studies of the interaction of sperm with the ZP [25,26], with a recent application of RFT to observations of monkey sperm supporting the idea that hyperactivation induces greater forces at the ZP [4].

However, the roles of mechanics, adhesion and flagellar beating in sperm–epithelium binding and sperm–ZP interactions have only been considered separately in modelling studies, even though the mechanical relevance of hyperactivation has been suggested by many researchers, and sperm binding is important for both processes. Hence, the differences in sperm mechanics that influences epithelial detachment on the one hand, and sperm–ZP interactions on the other hand, have not been explored. Furthermore, neither improvements beyond the limited accuracy of RFT nor the impact of cumulus elasticity have been considered in modelling sperm–ZP interactions, even though the latter would be supported by a now extensive theory for swimming in viscoelastic media [27–31]. Finally, the impact of adhesion in modelling investigations has been limited to the above-mentioned study of sperm–epithelial interactions by Simons *et al.* [21] and also has not been considered in the context of sperm encountering the ZP.

Hence, in this study, we extend the adhesive dynamics implemented by Simons *et al.* [21] to consider a fully three-dimensional movement of the cell incorporating the effect of a faithful sperm head geometry. This is implemented to numerically investigate the mechanics of sperm binding and behaviour for several observed flagellar waveforms via a direct numerical solver, the boundary element method (BEM) [22]. Our first aim is to examine sperm behaviour on encountering an adhesive substrate, and how this depends on the direction of sperm approach and the detailed binding dynamics, together with the flagellar waveform, especially whether or not it is hyperactivated. In particular, we examine the conditions under which sperm adhere to or escape from the substrate, how long they remain near the substrate when they do not adhere and the behaviour of the forces the sperm exerts on the substrate, including the impact of media elasticity. As such, we thus aim to improve our understanding of the interactions of sperm with an adhesive substrate. In particular, we assess whether our current mechanical understanding is consistent with the differing behaviours between sperm release from epithelial reservoirs and sperm behaviour at the ZP and, more generally, how hyperactivation and rheology may tune sperm behaviour near adhesive substrates.

## 2. Models and methods

### 2.1. Cell geometry and waveform

**Figure 2.**
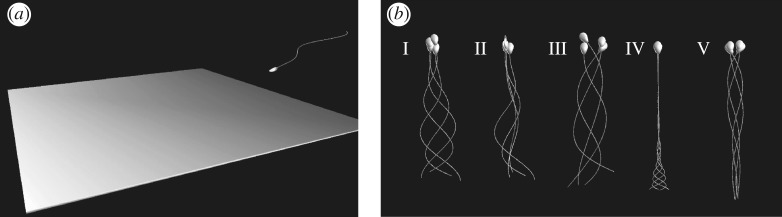
(*a*) A snapshot of the numerical simulation for a swimming spermatozoon near a rigid wall. (*b*) Snapshots of swimming spermatozoa with the five different beat patterns: (I) the non-hyperactivated planar beat, (II) the non-hyperactivated helical beat, and the hyperactivated beat within (III) a low-viscosity, water-like medium, (IV) a high-viscosity medium and (V) COC (cumulus) matrix. Four snapshots during one beat period with an equal time separation are superimposed and the analytical definitions of the waveforms are presented in the electronic supplementary material.

As illustrated in figure 2*a*, the model spermatozoon consists of a rigid model human sperm head, which is a deformation of an ellipsoid of dimension 1.1 × 2.8 × 4.5 µm, as used in previous studies [22,32]. In addition, the flagellum is a cylinder of length *L* = 56 µm based on human sperm dimensions [33], with a radius of *a* = 0.125 µm, and further details on these geometrical parameters are presented in the electronic supplementary material.

We consider five types of waveform, with snapshots illustrated in figure 2*b*. In particular, beat forms I and II are non-hyperactivated planar and helical beats, respectively, with primary observations from Dresdner *et al.* [34] and Suarez *et al.* [10]. Furthermore, beat pattern I is planar and symmetric, and is used as a reference waveform, whereas waveform II introduces helicity into what is otherwise the reference beat pattern. Beat patterns III, IV, V, respectively, correspond to hyperactivation in a low-viscosity medium, a simple high-viscosity medium such as methylcellulose solution and the COC matrix, with primary observations for the waveforms taken from several studies [5,10,35–37]. In particular, hyperactivated beat pattern III is planar, with asymmetry and a low wavenumber and is observed in watery media. Beat pattern IV, for hyperactivated sperm in a high-viscosity medium, exhibits a very low beat frequency and a suppression of proximal beating, whereas beat pattern V, for hyperactivated sperm in cumulus, also has a very low beat frequency but, in contrast, a suppression of distal bending.

### 2.2. Wall interaction

We use a rigid smooth wall, on which the no-slip boundary condition is satisfied, to model the surface of the oviductal epithelium and also the egg ZP, and thus neglect surface topography and cilia on the tract epithelium, and also curvature of the egg. There are no quantitative data on the interaction between a sperm and a substrate, though cell–wall interactions are reported up to a scale of 100 nm from a substrate for *Escherichia coli* [38]. This interaction can be attractive or repulsive according to the solutes present [38], as indicated for sperm by the use of albumin to prevent substrate sticking [33]; below, we treat this interaction as repulsive, so that sperm can stably swim above the substrate. We also further note that sperm adhesion to the ZP and female reproductive tract epithelia is regularly observed [39] and thus we also include adhesion mechanics below.

Hence, first and as used in a previous study [32], we introduce a repulsive interaction potential with the wall repulsive force per unit area of the cell surface at the position ***x*** given via2.1
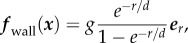
where *r* is the distance from the surface and ***e**_r_* denotes the outward normal of the wall body. The constants, *g* = 200 µω and *d* = *L*/500 ≈ 0.11 µm, entail a very strong repulsion, greater than other scales in the model, once the sperm is within about 100 nm of the surface.

We proceed to represent sperm–wall binding using an elastic bond, as detailed and motivated by Simons *et al.* [21], with bonds forming at each head surface position when the distance from the wall is closer than *R*_on_. With **e***_r_* the unit vector in direction from the bond location on the wall to its bonded counterpart on the sperm head, then for a point located at ***x*** on the head surface, the length of the bond is given by the modulus of
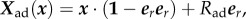
where **1** denotes the identity tensor, and the adhesion force is assumed to be that of a linear spring, whereby
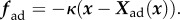


However, the bond is broken and removed, once the bond length exceeds the length *R*_off_. Finally, note that linkages are taken to only form between the substrate and the sperm head, and not the sperm flagellum. The associated parameter values are summarized in table 1.

**Table 1.** Reference parameters and scales for the sperm model and cell–substrate interaction models, which are used throughout this study; further details on the flagellum radius, *a*, can be found in the electronic supplementary material. The fluid viscosity *μ*_0_ is (essentially) that of water.

parameter	interpretation	value	reference
*a*	flagellar radius	0.125 µm	[18]
*L*	flagellar length	56 µm	[18]
*μ*_0_	fluid viscosity (beat patterns I,II,III of figure 2*b*)	0.001 Pa s	
*μ*	fluid viscosity (beat pattern IV of figure 2*b*)	4000*μ*_0_	[10]
*μ*	fluid viscosity (beat pattern V of figure 2*b*)	100*μ*_0_	[37]
*ω*_0_	beat frequency (beat pattern I, II of figure 2*b*)	40*π* Hz	[5]
*ω*	beat frequency (respectively beat pattern III, IV, V of figure 2*b*)	0.5*ω*_0_, 0.2*ω*_0_, 0.3*ω*_0_	[5,33,37]
*g*	wall repulsion strength	200*μω*	[32]
*d*	wall repulsion length	0.002*L*	[32]
*R*_ad_	adhesion model parameter	0.01*L*	[21]
*R*_on_	adhesion model parameter	0.0101*L*	[21]
*R*_off_	adhesion model parameter	0.011*L*	[21]

### 2.3. Fluid dynamics

Sperm swim at negligible Reynolds number with the momentum balance given by2.2
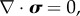
where 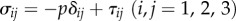 for a pressure field *p* and a deviatoric stress tensor ***τ***. In a Newtonian fluid, one has 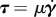, where2.3
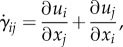
with *μ* the constant viscosity of the fluid and with ***u*** denoting the flow velocity, which also satisfies the incompressibility condition, 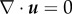.

We impose no-slip boundary conditions on the wall and, given the sperm waveform, the cell surface as well. Closing the system with the force and torque balance equations for the cell body, the swimming velocity of the model spermatozoon can be obtained. The resulting linear equations are conveniently expressed and solved by the BEM, as detailed in the electronic supplementary material.

We also consider a linear viscoelastic Maxwell fluid, where the deviatoric stress tensor is given by2.4
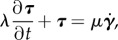
which approximates the flow properties of media such as methylcellulose solutions [33], where *λ* is the elastic relaxation time. For a sperm adhered to the wall, with no rotation about the contact point, the force it exerts on the surface is given by2.5
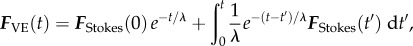
where ***F***_Stokes_(*t*) is the force exerted by a similarly surface-adhered spermatozoon in a Newtonian fluid of the same viscosity *μ*, and can be calculated using the BEM, as derived in the electronic supplementary material.

## 3. Results I: low-viscosity medium

**Figure 3.**
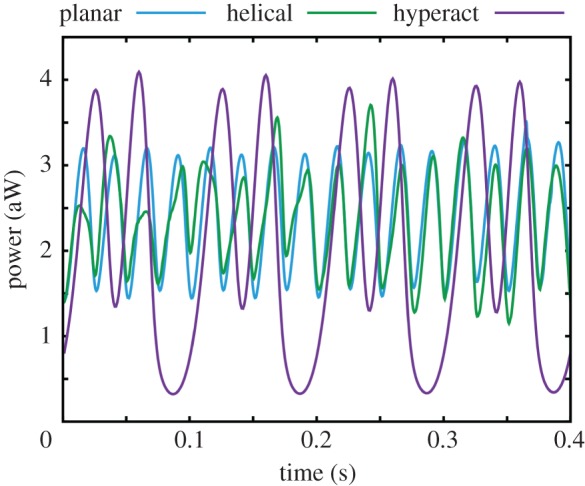
The mechanical power consumption for a spermatozoon swimming within a low-viscosity medium, with waveforms I, II, III of figure 2*b*, in units of attowatts.

First, we investigate the boundary behaviours of spermatozoa surrounded by a water-like medium with a low viscosity, *μ*_0_; hence, we first focus on waveforms I, II and III in figure 2*b*. Using numerical techniques discussed in the electronic supplementary material, we compute the power output [40]
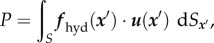
where ***f***_hyd_ is the hydrodynamic surface traction on the cell surface and ***u*** is the velocity field; this reveals that there is no substantial power difference for waveforms I–III, as shown in figure 3. In turn, this is consistent with the observation that the cell energy expenditure does not significantly change before and after hyperactivation [5].

It is helpful in classifying the results below to introduce the non-dimensional parameter3.1
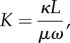
where *μ* is the viscosity and *ω* is the flagellar beat frequency—for planar beating in watery media, as here, these are *μ*_0_ and *ω*_0_ of table 1. The parameter *K* represents the relative strength of the wall adhesion compared with the hydrodynamic viscous force, hereafter referred to as the *bond strength*, and this becomes the same order of magnitude as that used in Simons *et al.* [21] once *K* ≈ 10^5^.

### 3.1. Planar waveform

We proceed to model a sperm swimming near a wall incorporating the repulsive potential and adhesive bonds between the head and substrate. First, the impact of the initial orientation of the sperm relative to the substrate is considered, given a planar waveform and the absence of hyperactivation; the latter, in particular, entails that the planar waveform (beat I) in figure 2*b* is used.

We consider spermatozoa initially located with the head–flagellum junction a distance 0.3*L* above the wall with an initial angle of attack given by 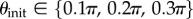 and with the beat plane oriented as shown in figure 2*a*, for the parameters given by table 1 and the bond strength fixed at *K* = 10^4^.

**Figure 4.**
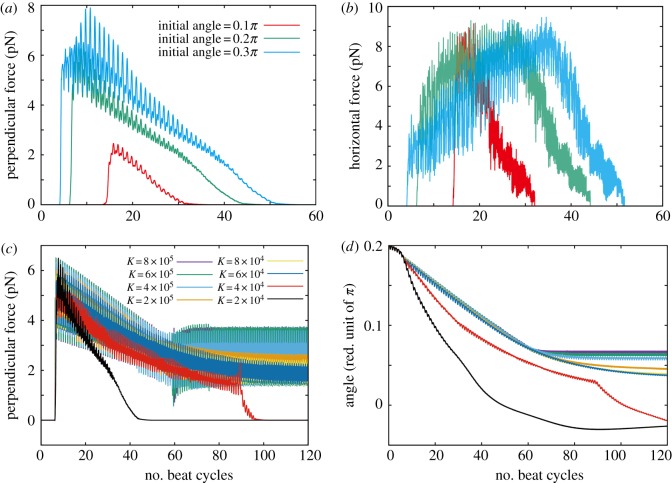
Results from the numerical simulation of a sperm with the planar waveform I of figure 2*b*. (*a*) The computed perpendicular force upon the surface with initial angles *θ*_init_ = 0.1*π*, 0.2*π*, 0.3*π*, and the bond strength fixed at *K* = 10^4^; all these sperm ultimately escape the surface owing to the low bond strength. (*b*) The absolute value of the horizontal force acting on the wall for the sperm in plot (*a*). The simulation parameters and the colours of the plots are the same as in (*a*). (*c*) The perpendicular force upon the wall for the different bond strengths and initial angle *θ*_init_ = 0.2*π*. (*d*) The time evolution of the angle towards the wall. The parameters and the colour of the plots are the same as in (*c*), with a positive angle corresponding to the sperm oriented towards the substrate.

In figure 4*a*,*b*, there are plots of the components of the force exerted by the swimming sperm on the wall which, for each cell, is given by
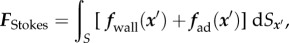
where *S* is the surface of both the sperm head and flagellum. The perpendicular component of ***F***_Stokes_ increases with the angle of attack (figure 4*a*), but in every simulation, the cell head gradually aligns along the wall (electronic supplementary material, movie S1), so that this perpendicular force ultimately decreases. The cell eventually breaks away from its wall interaction, once the force exerted by the sperm along its bonds, which is essentially the magnitude of the parallel (horizontal) component, exceeds the load limit of the bonds, approximately 9 pN for a near horizontal configuration, as can be seen in figure 4*b* for escaping cells.

Further simulations demonstrate that the initial virtual sperm angle of attack has no significant influence on the large time dynamics, though the size of the interaction forces and the time adhered can vary. Thus, hereafter we fix the initial angle of attack to be *θ*_init_ = 0.2*π*, and examine the impact of the bond strength on sperm behaviour in figure 4*c*,*d*. These respectively plot the time evolution of the perpendicular force exerted by the sperm on the wall and the angle of the sperm relative to the wall for simulations with different values of the bond strength, *K*. In all cases, the angle of the sperm relative to the wall decreases as time progresses, which, in turn, decreases the perpendicular force upon the wall (electronic supplementary material, movie S1). For lower bond strengths, this angle eventually becomes negative, corresponding to detachment from the wall. After this separation, the cell never returns to wall proximity.

**Figure 5.**
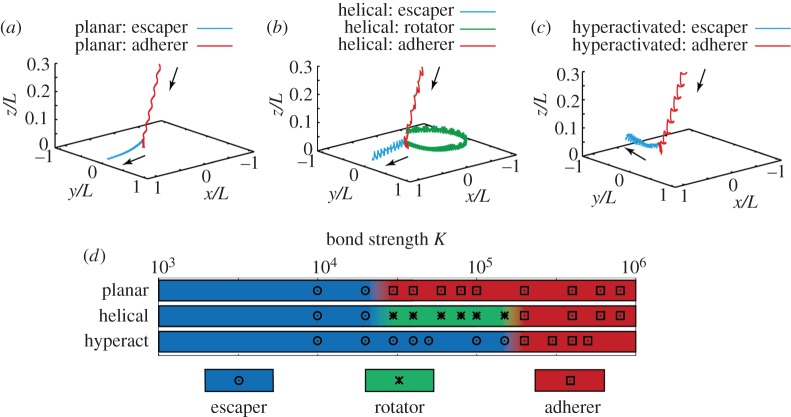
The computed trajectories of the sperm head–tail junction for the three low-viscosity waveforms depicted in figure 2*b*, together with a summary of the long-time sperm behaviour for the different waveforms as the bond strength is varied. (*a*) Trajectories for a sperm with the planar waveform, beat pattern I of figure 2*b*, with a bond strength of *K* = 10^4^ (escaper) and *K* = 10^5^ (adherer). (*b*) Trajectories of sperm with the helical waveform, pattern II of figure 2*b*, for three different bond strengths: *K* = 10^4^ (escaper), *K* = 10^5^ (rotator) and *K* = 10^6^ (adherer). (*c*) Trajectories of the sperm with the hyperactivated waveform, pattern III of figure 2*b*, for bond strengths of *K* = 10^5^ (escaper) and *K* = 10^6^ (adherer). (*d*) The long-time behaviour of sperm is depicted by colour for each of the given beat patterns (I, planar; II, helical; or III, hyperactivated) and for a spectrum of bond strengths, with the regions between the different final cell behaviours illustrated by a transition of the colours. In particular, these transitions simply reflect that the long-time behaviour changes somewhere in this region, because only a discrete number of bond strengths have been simulated.

In figure 5*a*, two representative trajectories are illustrated in detail for planar beaters, for *K* = 10^4^, where the cell escapes from the adhesion interaction, and for *K* = 10^5^, where the sperm adheres, and these two outcomes are representative of all observed behaviours for the planar beaters, as can be seen in figure 5*d*. Further, with a sufficiently large bond strength, the cell fails to detach from the wall and is captured by the adhesive attraction. In this case, the sperm continuously applies a force to the wall and orients to a small, positive angle of attack. In the time sequences of the force (figure 4*c*) with larger bond strengths, a sharp change is observed around 60 beat cycles in the simulation, which corresponds to the initiation of the plateauing of the angle in figure 4*d*, and an attraction to the stable configuration of the sperm head, which is accompanied by the creation of the large number of adhesive bonds. In particular, the simulations for sperm with the planar waveform and adhesive dynamics indicate that whether or not detachment from the wall occurs is governed by the competition between the force exerted on the wall by sperm, which is effectively horizontal, and the total bond strength once the sperm head is nearly aligned along the wall.

### 3.2. Helical waveform

For helical beating in the absence of hyperactivation, i.e. waveform II of figure 2*b*, the long-time sperm dynamics is not affected by its initial angle of orientation, as with the planar beater. In figure 5*b*, the trajectories of the cell head–tail junction given a helical flagellar beat pattern with no hyperactivation are depicted for the bond strengths *K* = 10^4^, 10^5^, 10^6^. The parameters used in the simulations are presented in table 1, and the initial angle of attack is *θ*_init_ = 0.2*π*. At the lower bond strength, the cell overcomes the attractive force owing to the adhesive bonds. Nevertheless, despite breaking the adhesion bonds to the extent it can move, the cell swims within 3–4 µm from the wall, which would facilitate sperm rheotaxis, that is upstream swimming owing to a background flow [41].

Increasing the bond strength to *K* = 10^5^ induces a cell cycling movement across the substrate, with a novel adhesive dynamics that is neither a complete detachment from the wall nor a stable and fixed adhesion, as shown by the green trajectory in figure 5*b* and also electronic supplementary material, movie S2. The torque generated by the helical flagellar beat detaches one end of the sperm head, whereas the other end is adhered to the wall. After this detachment, the head rotates about its adhesions whereby, after a half rotation of the head, it eventually fully adheres once more. This dynamics persists, leading to a continuous head cycling movement across a relatively small region of the surface though total detachment does not occur. With a further increase in the bond strength, the torque, owing to the flagellar movement, no longer exceeds the threshold of the adhesive attractions, and the cell is thus stably fixed at one point of the substrate, as shown by the red trajectory in figure 5*b*.

### 3.3. Hyperactivated waveform in low-viscosity medium

We now explore the impact of hyperactivation on sperm adhesion dynamics, using the asymmetric waveform of beat III in figure 2*b* and, once more, the initial sperm head orientation does not influence the long time dynamics. We therefore use *θ*_init_ = 0.2*π*, and observe in figure 5*c* that the hyperactivated sperm adheres or escapes according to bond strength, with the latter demonstrated in electronic supplementary material, movie S3. With detachment, longer time simulations (not presented) show that the hyperactivated sperm with beat pattern III swims away from the wall, to distances greater than 300 µm with a curved trajectory reflecting the beat pattern asymmetry, and eventually returns to the proximity of the wall though it never adheres.

In figure 5*d*, the final sperm behaviour is surveyed for each of the three types of waveforms in a low-viscosity medium as the bond strength is varied, with parameters given in table 1. Three behaviours are seen: detachment (an escaper cell), stable and fixed adhesion (an adherer cell) and finally the rotational motion at the surface exhibited by the helical beater (a rotator cell), which are indicated by blue, red and green, respectively. With *K* = 10^5^, the planar beater is adhered, the helical beater exhibits a rotator behaviour, which is still constrained to the surface, whereas the hyperactivated sperm is an escaper. This highlights that hyperactivation is predicted to enhance surface detachment and also that about 10 times the bond strength is required to keep the hyperactive sperm attached to the surface, compared with the planar waveform swimmer, even though the power expenditure of all three beat patterns is broadly the same.

This overall trend was also observed to be unaffected by changing the head geometry to that of a sphere of the same volume, or by increasing the disconnection length of the bond linkage, *R*_off_ by close to a factor of two, as evidenced in the electronic supplementary material, highlighting the robustness of predicted large time sperm behaviours to model parameters.

## 4. Results II: a comparison of low- and high-viscosity media and consideration of the zona pellucida

### 4.1. Low-viscosity medium

**Figure 6.**
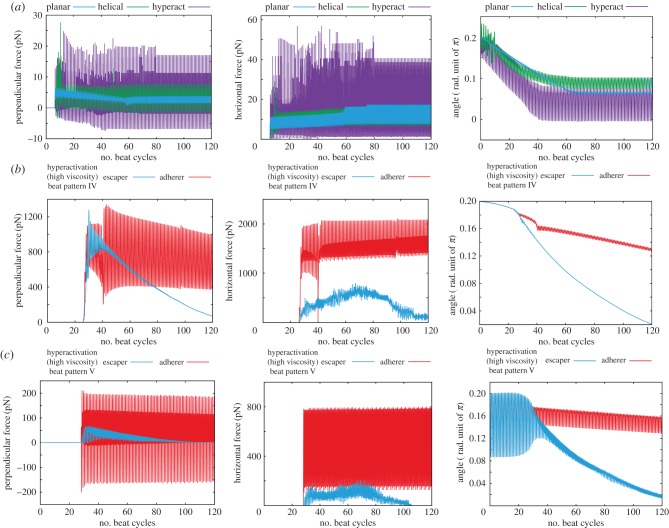
Time evolution of the predicted force exerted by a spermatozoon and its angle towards the surface, for simulations with different flagellar waveforms, as depicted in figure 2*b*. (*a*) Results for a spermatozoon with a binding strength of *K* = 5 × 10^5^ and a waveform associated with a low-viscosity medium, respectively, a planar beat pattern, a helical beat pattern and a hyperactivated beat pattern (waveforms I, II, III of figure 2*b*). (*b*) Results for a spermatozoon with a beat pattern associated with a high-viscosity medium such as methylcellulose solution, waveform IV of figure 2*b*, and with two binding strengths, *K* = 1 × 10^6^ (escaper) and 1 × 10^7^ (adherer). (*c*) Results for a spermatozoon with a beat pattern associated with cumulus, waveform V of figure 2*b*, with two binding strengths, *K* = 3 × 10^5^ (escaper) and 1 × 10^7^ (adherer). The force perpendicular to the surface (*left-hand column*) is taken to be positive when the spermatozoon is repelled from the surface. In the *central column*, the magnitude of the surface interaction force parallel to the surface is presented. In the *right-hand column*, the angle between the surface and the spermatozoon is given, with positive values corresponding to the head of the spermatozoon oriented towards the wall.

For sperm with lower viscosity waveforms near a wall and a larger bond strength, *K* = 5 × 10^5^, that induces adherence, the time sequences of the perpendicular and the horizontal components of the force exerted on the substrate by the sperm are plotted in figure 6*a* together with the angle of the sperm relative to the wall. For the two non-hyperactivated flagella beat patterns, I and II of figure 2*b*, the perpendicular force exerted by the spermatozoon decreases with adhesion initiation, whereas the horizontal force gradually increases. Stable adhesion is eventually achieved with a nearly stationary and small angle of orientation of the sperm head relative to the surface. In contrast, a sperm with the hyperactivated waveform III of figure 2*b* induces more extensive oscillations and larger peak forces, approximately 15 and 40 pN perpendicular and parallel to the surface, respectively, about two to three times larger than the peak forces exerted in the absence of hyperactivation, which are about 5–8 and 15 pN in respective comparison.

### 4.2. High-viscosity medium

Within a highly viscous medium, the hyperactivated beat pattern changes, as reflected by beat IV of figure 2*b* for a methylcellulose solution [10] or beat V for cumulus [35]. Simulations with these waveforms, and the parameters of table 1, are presented in electronic supplementary material, movies S4 and S5, respectively, with an initial angle of attack given by *θ*_init_ = 0.2*π*. The initial cell orientation relative to the surface once more has no influence on the ultimate sperm behaviour, which is either that of the escaper or adherer. The critical bond strength for the transition between these behaviours is approximately *K* = 4 × 10^6^ for beat IV and 6 × 10^6^ for beat V, which are slightly larger than the transitional bond strength for beat patterns associated with low-viscosity medium.

In figure 6*b*,*c*, the time sequences for the forces exerted by the virtual sperm on the wall and its angle relative to the wall are presented for simulations with beat patterns IV and V of figure 2*b*. For the former, the bond strengths are approximately *K* = 1 × 10^6^ and *K* = 1 × 10^7^ in the simulations, whereas for the latter, bond strengths of approximately *K* = 3 × 10^5^ and *K* = 1 × 10^7^ are used, with all other parameters given by table 1 and the same initial conditions used in §4.1.

Regardless of the final virtual sperm behaviour, the perpendicular peak force exerted by the cell on the wall with beat pattern IV exceeds 1000 pN, which is 50 times larger than the peak force generated by the hyperactivated flagellum in low-viscosity medium. This therefore indicates the enhancement of the sperm penetrative potential owing to increased viscosity of the surrounding medium. This enhancement is also found in the plots for beat V (figure 6*c*), where the perpendicular force is increased to 200 pN, though the force also oscillates in time and becomes negative during a beat cycle.

Furthermore, in the absence of a sufficiently strong bond affinity for adhesion, the timescale for the duration of the interaction between the sperm and the surface is approximately 20 s for beat pattern IV and 10 s for waveform V. These timescales are much longer than the predicted wall–sperm interaction duration for the hyperactivated waveform in the low-viscosity medium, which is approximately 1 s for weak adhesions (figure not shown). It should also be noted that decreasing the bond strength reveals no significant change in the peak of the force or, if the sperm does not permanently adhere, the duration of interaction that additionally highlights that the viscous medium is predicted to enhance the potential for sperm to interact with the ZP even with weak surface affinity.

### 4.3. The impact of elasticity on the interaction force between the virtual sperm and the wall

**Figure 7.**
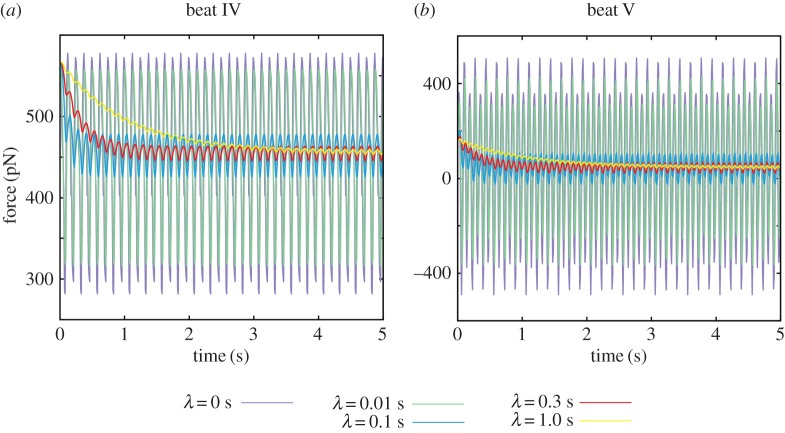
The time evolution of the perpendicular force exerted by a spermatozoon on a wall given its head is adhered with no rotation and the flagellum is beating in a linearly viscoelastic Maxwell fluid, with elastic relaxation time *λ*. (*a*) Results for beat IV of figure 2*b*. (*b*) Analogous results for beat V.

The perpendicular force exerted by a spermatozoon in a viscoelastic medium is computed via equation (2.5) for waveforms IV and V of figure 2*b* with a range of the elastic relaxation times, *λ*, and plotted in figure 7. The sperm head is rigidly fixed, as required for the validity of equation (2.5), and corresponds to the large bond strength limit, i.e. *K* large. The angle between the cell and the surface is set to 0.1*π* and 0.05*π* for beat IV and beat V, which are the long time values for Newtonian simulations of adhered sperm, though the effect of elasticity is not sensitive to the details of this angle.

To proceed, it is convenient to introduce the Deborah number, *De* = *λ*/*T* = 2*πλω*, which estimates the relative importance of elasticity compared with viscosity for the medium [27]. The characteristic relaxation time on which the viscous and the elastic properties are balanced, i.e. *De* approximately 1, is given by *λ_c_* ≈ 0.25 s for beat IV and *λ_c_* ≈ 0.17 s for beat V, respectively. The perpendicular force exerted by the sperm on the wall tends to ≈450 pN for beat pattern IV and to ≈50 pN for beat pattern V for a large elastic relaxation time *λ* and such forces are still much larger than the forces exerted on the wall by a spermatozoon in a low-viscosity Newtonian medium. It should be noted that the sign of the force for beat V oscillates for a weakly elastic fluid, but the force becomes positive in a medium with high elasticity, which also reflects the material properties of cumulus [9].

## 5. Discussion

We have numerically investigated sperm swimming and sperm head–surface adhesion dynamics in the vicinity of a rigid flat surface using observed flagellar waveforms [10,34–36]. To the extent that the variable human sperm morphology allows [42], a geometrically faithful human sperm head has been used in the simulations and our objective is to provide further mechanical understanding for both the sperm–epithelium and the sperm–ZP interactions and adhesion, especially in the context of the mechanical impact of hyperactivation and also media rheology.

Via numerous simulations with repulsive surface potential and binding adhesions, it has been observed that local mechanics between the sperm cell head and the surface dictates surface behaviour, with the cell aligning essentially along the surface, regardless of orientation when adhesion initiates and the ultimate outcome of the cell–surface interaction. Furthermore, although the mechanical power of the flagellar bending does not change significantly with hyperactivation in a low-viscosity, water-like, medium, the hyperactivated waveform is predicted to require bonding strengths which are about 10 times larger to adhere the cell to the surface, compared with a non-hyperactivated cell. In addition, the peak force acting on the surface is increased two to three times for the hyperactivated beat compared with the non-hyperactivated beat in a low-viscosity medium. The latter force is approximately 5–8 pN in the perpendicular direction and broadly comparable to estimates reported in the literature, as determined by the relatively crude approximation of RFT, which are in the range of 4–19 pN [25,26,43], even though the previous formulations are highly simplified and the detailed computational situations differ.

Such modelling observations further support the prospect that hyperactivation substantially contributes to detachment from the isthmic epithelium, as concluded for planar beating flagella without cell bodies in previous modelling studies [20,21]. One should also note that the oscillation in the sign of the force exerted on the surface, periodically acting to pull the sperm away from the surface, requires the hyperactivated waveform to have an element of proximal bending as in the hyperactivated beat pattern III of figure 2*b* in contrast to beat pattern IV, corresponding to hyperactivated sperm in *in vitro* methylcellulose solutions [10]. However, proximal bending is still observed *in vivo* during epithelial sperm detachment [8] and hence the former beat pattern is used for considering isthmic oviductal sperm detachment.

A novel surface dynamics is observed for virtual sperm with the helical waveform, given a tuned parameter regime, inducing sperm head circling on the surface without detachment. Such behaviour also would require an appropriate choice of sperm species and viscosity to maintain helical waves, with Woolley [44] reporting that three-dimensional flagellar beating can be suppressed near surfaces, though this is not universal [45,46]. While such dynamics does not appear to be common, and is predicted to require stringent restrictions, mouse sperm can be observed to tightly cycle on a surface before detaching [47], which shares similar dynamical features to the rolling motility predicted here.

Hyperactivation is required for penetrating the cumulus, as reported for mice [13]. More generally, in a highly viscous medium, the hyperactivated sperm waveform is observed to become straighter, with only the distal part of the flagellum bending in *in vitro* methylcellulose solutions [10] and, in contrast, only the proximal part of the flagellum bending in the cumulus [35]. Hence, we have considered the impact of such beat patterns as sperm approach and adhere to a substrate within a highly viscous medium and also how this contrasts for a low-viscosity medium, which are often used for *in vitro* studies of sperm–egg interactions.

In the context of contact with the ZP, we have the ubiquitous modelling observation that sperm heads align with the surface and thus sperm penetration initiates nearly tangentially to the egg surface, as documented by Green & Purves [48]. Furthermore, without a sufficiently strong affinity between the head and the surface, the predicted duration of surface proximity is typically ≈1 s for the hyperactivated beat pattern associated with a low-viscosity medium, with an initial angle of attack given by *θ*_init_ = 0.2*π*. This is much shorter than the analogous duration for hyperactivated waveforms associated with highly viscous media, which suggests the prospect of reduced proximity with, and possible penetration of, the ZP in a cumulus-free surrounding when there is insufficient binding, as observed in aberrant zona-binding mice sperm *in vitro* [2].

The modelling also predicts an extensive, order of magnitude increase or more, in the force exerted by the sperm on the substrate for the cumulus beat pattern, waveform V of figure 2*b*, though such predictions are contingent on a reliable estimate of the material properties. While standard macroscopic rheology measures may not be appropriate, due to the fact sperm swim on a microrheological lengthscale, Drobnis *et al.* [35] use RFT to estimate the cumulus viscosity from observations of hamster sperm, thus generating a viscosity estimate relevant for the lengthscale of sperm of *μ* ≈ 0.1–0.4 Pa s. While further caution is required as the cumulus is gradually dissolved during the fertilization process, and thus its viscosity reduces in time [49] and is highly variable, overall these simulations are indicative of an increased force on a surface when a hyperactivated sperm adheres to a surface while surrounded by cumulus compared with a watery medium. Furthermore, reducing the binding strength also does not significantly alter the predicted forces, emphasizing that the high viscosity beat pattern induced by the cumulus is predicted to have the potential of enhancing ZP penetrative ability and this is regardless of the details of the adhesion bond strength, given adhesion does occur.

Further analysis of the force exerted on a substrate by a hyperactivated spermatozoon has been performed by considering a linearly viscoelastic fluid, with linear elasticity motivated by the microscopic lengthscale of the sperm flagellum waveform amplitude, together with the observation that cumulus is mechanically linear for deformations on much larger lengthscales [9]. The elastic relaxation time is estimated to be ≈0.02 s for 2% methylcellulose solution [33], and ≈4 s for polyacrylamide solution in a corn syrup [50], and the relaxation time for cervical mucus is predicted to be of the order of 1–10 s [27]. Because the cumulus is reported to be highly elastic [9], it is expected to have a relatively large relaxation time. From our results for a viscoelastic medium with a hyperactviated sperm clamped by a large bond strength, we found the oscillations in the forces exerted by the sperm on the substrate are damped and phase lagged relative to the Newtonian case with longer relaxation times, as illustrated for the perpendicular force. Furthermore, this perpendicular force becomes uniformly positive with increasing elasticity for beat pattern V, which is associated with sperm behaviour in the cumulus. Such modelling observations generate the prediction that high cumulus elasticity induces sperm to push into the ZP, in contrast to the oscillating perpendicular force associated with isthmic oviductal sperm detachment, discussed earlier. Hence, the presence of the cumulus is not only predicted to increase the forces exerted by the sperm, even with the damping associated with high elasticity, but also to consistently direct the sperm to push into the ZP.

Furthermore, extensive *in vitro* studies report that sperm without ZP binding ability are infertile *in vitro* but can successfully fertilize eggs surrounded with cumulus [14–16] and this effect of the cumulus has been described as a ‘mystery’ in the mechanism of fertilization [51,52]. Our results for sperm dynamics near adhering surfaces support a prospective explanation, with predictions of an extensive increase in both the sperm force and also the duration of sperm proximity to the egg owing to mechanical changes and altered beat patterns associated with the cumulus. In turn, this supports the hypothesis that the cumulus provides mechanical facilitation [35], compensating for an insufficiency of the sperm mechanical forces alone [53] for zona penetration.

Finally, note that, as detailed in the electronic supplementary material, the qualitative details of the predictions for the virtual sperm are not sensitive to the head geometry, with analogous remarks for changes in the parameter sets for binding dynamics, flagellar lengthscales and waveform timescales. Thus, one may anticipate that the qualitative conclusions drawn above are applicable across many substrates and many species of mammalian spermatozoa. In addition, there is no sensitivity in the cell adhesion dynamics with respect to surface curvature when considering predicted sperm behaviours near a sphere with a typical human egg cell radius. This is confirmed by simulations in the electronic supplementary material and is consistent with the above results, that adhesion or detachment is determined by the local mechanics on the scale of a sperm head.

In summary, this modelling study of sperm dynamics near a substrate with adhesive bindings has revealed multiple mechanical functions of hyperactivation. For instance, there is an enhancement of detachment from the isthmic epithelium, and an amplification of the sperm forces when surrounded by a highly viscous medium. Furthermore, even with weak surface adherence, highly viscous media significantly increase the duration for which a hyperactivated sperm remains near the surface. In addition, high medium elasticity that reflects the cumulus induces a constant pushing of hyperactivated sperm into the substrate indicating a potential difference in behaviour for hyperactivated sperm unbinding at the isthmic epithelium compared with sperm pushing at the ZP. More generally, by controlling the waveform and the surrounding medium, this modelling study predicts that sperm behaviour near an adhesive substrate can be tuned, and thus that there is a prospective role for mechanics in explaining differing sperm behaviours near, or attached to, different physiological substrates and within different media.

## Supplementary Material

Supplementary Materials

## References

[1] Suarez SS, Pacey AA 2006 Sperm transport in the female reproductive tract. Hum. Reprod. Update 12, 23–37. (10.1093/humupd/dmi047) 16272225

[2] Okabe M 2013 The cell biology of mammalian fertilization. Development 140, 4471–4479. (10.1242/dev.090613) 24194470

[3] Yanagimachi R 1970 The movement of golden hamster spermatozoa before and after capacitation. Reproduction 23, 193–196. (10.1530/jrf.0.0230193) 5472441

[4] Ishijima S 2011 Dynamics of flagellar force generated by a hyperactivated spermatozoon. Reproduction 142, 409–415. (10.1530/REP-10-0445) 21670125

[5] Ooi EH, Smith DJ, Gadêlha H, Gaffney EA, Kirkman-Brown J 2014 The mechanics of hyperactivation in adhered human sperm. R. Soc. open sci. 1, 140230 (10.1098/rsos.140230) 26064546 PMC4448887

[6] Morales P, Roco M, Vigil P 1993 Human cervical mucus: relationship between biochemical characteristics and ability to allow migration of spermatozoa. Hum. Reprod. 8, 78–83. 8458932 10.1093/oxfordjournals.humrep.a137879

[7] Ho K, Wolff CA, Suarez SS 2009 Catsper-null mutant spermatozoa are unable to ascend beyond the oviductal reservoir. Reprod. Fertil. Dev. 21, 345–350. (10.1071/RD08183) 19210926

[8] Chang H, Suarez SS 2012 Unexpected flagellar movement patterns and epithelial binding behavior of mouse sperm in the oviduct. Biol. Reprod. 86, 140 (10.1095/biolreprod.111.096578) 22337334 PMC3364923

[9] Dunn PF, Picologlou DF 1976 Viscoelastic properties of cumulus oophorus. Biorheology 13, 379–386. 1009242 10.3233/bir-1976-13605

[10] Suarez SS, Dai X 1992 Hyperactivation enhances mouse sperm capacity for penetrating viscoelastic media. Biol. Reprod. 46, 686–691. (10.1095/biolreprod46.4.686) 1576267

[11] Baba D, Kashiwabara S, Honda A, Yamagata K, Wu Q, Ikawa M, Okabe M, Baba T 2002 Mouse sperm lacking cell surface hyaluronidase PH 20 can pass through the layer of cumulus cells and fertilize the egg. J. Biol. Chem. 277, 30 310–30 314. (10.1074/jbc.M204596200) 12065596

[12] Yanagimachi R 1981 Mechanisms of fertilization in mammals. In Fertilization and embryonic development in vitro (eds Mastroianni L Jr, Biggers JD), pp. 81–182. Berlin, Germany: Springer.

[13] Quill TA, Sugden SA, Rossi KL, Doolittle LK, Hammer RE, Garbers DL 2003 Hyperactivated sperm motility driven by CatSper2 is required for fertilization. Proc. Natl Acad. Sci. USA 100, 14 869–14 874. (10.1073/pnas.2136654100) PMC299835 14657366

[14] Nishimura H, Nakanishi T, Kim E, Baba T 2004 Possible function of the adam1a/adam2 fertilin complex in the appearance of adam3 on the sperm surface. J. Biol. Chem. 33, 34 957–34 962. (10.1074/jbc.M314249200) 15194697

[15] Inoue N, Satouh Y, Okabe M, Ikawa M, Yanagimachi R 2011 Acrosome-reacted mouse spermatozoa recovered from the previtelline space can fertilize other eggs. Proc. Natl Acad. Sci. USA 108, 20 008–20 011. (10.1073/pnas.1116965108) PMC3250175 22084105

[16] Tokuhiro K, Ikawa M, Benhama AM, Okabe M 2012 Protein disulfide isomerase homolog pdilt is required for quality control of sperm membrane protein adam3 and male fertility. Proc. Natl Acad. Sci. USA 109, 3850–3855. (10.1073/pnas.1117963109) 22357757 PMC3309714

[17] Fauci LJ, McDonald A 1995 Sperm motility in the presence of boundaries. Bull. Math. Biol. 57, 679–699. (10.1016/s0092-8240(05)80768-2) 7606221

[18] Smith DJ, Gaffney EA, Blake JR, Kirkman-Brown JC 2009 Human sperm accumulation near surfaces: a simulation study. J. Fluid Mech. 621, 289–320. (10.1017/S0022112008004953)

[19] Elgeti J, Kaupp UB, Gompper G 2010 Hydrodynamics of sperm cells near surfaces. Biophys. J. 99, 1018–1026. (10.1016/j.bpj.2010.05.015) 20712984 PMC2920720

[20] Curtis MP, Kirkman-Brown JC, Connolly TJ, Gaffney EA 2012 Modelling a tethered mammalian sperm cell undergoing hyperactivation. J. Theor. Biol. 309, 1–10. (10.1016/j.jtbi.2012.05.035) 22727894

[21] Simons J, Olson S, Cortez R, Fauci L 2014 The dynamics of sperm detachment from epithelium in a coupled fluid-biochemical model of hyperactivated motility. J. Theor. Biol. 354, 81–94. (10.1016/j.jtbi.2014.03.024) 24685890

[22] Ishimoto K, Gaffney EA 2014 A study of spermatozoan swimming stability near a surface. J. Theor. Biol. 360, 187–199. (10.1016/j.jtbi.2014.06.034) 25014474

[23] Jikeli JF et al. 2009 Sperm navigation along helical paths in 3D chemoattractant landscapes. Nat. Commun. 6, 7985 (10.1038/ncomms8985) PMC4557273 26278469

[24] Gray J, Hancock GJ 1955 The propulsion of sea urchin spermatozoa. J. Exp. Biol. 32, 802–814.

[25] Baltz JM, Katz DF, Cone RA 1988 Mechanics of sperm–egg interaction at the zona pellucida. Biophys. J. 54, 643–654. (10.1016/S0006-3495(88)83000-5) 3224150 PMC1330369

[26] Green DPL 1988 Sperm thrusts and the problem of penetration. Biol. Rev. 63, 79–105. (10.1111/j.1469-185X.1988.tb00469.x) 3284599

[27] Fulford GR, Katz DF, Powell RL 1998 Swimming of spermatozoa in a linear viscoelastic fluid. Biorheology 35, 295–309. (10.1016/S0006-355X(99)80012-2) 10474656

[28] Fu HC, Powers TR, Wolgemuth CW 2007 Theory of swimming filaments in viscoelastic media. Phys. Rev. Lett. 99, 258101 (10.1103/PhysRevLett.99.258101) 18233558

[29] Fu HC, Wolgemuth CW, Powers TR 2008 Beating patterns of filaments in viscoelastic fluids. Phys. Rev. E 78, 041913 (10.1103/PhysRevE.78.041913) 18999461

[30] Teran J, Fauci L, Shelley M 2010 Viscoelastic fluid response can increase the speed and efficiency of a free swimmer. Phys. Rev. Lett. 104, 038101 (10.1103/PhysRevLett.104.038101) 20366685

[31] Li G, Ardekani AM 2015 Undulatory swimming in non-Newtoninan fluids. J. Fluid Mech. 784, R4 (10.1017/jfm.2015.595)

[32] Ishimoto K, Cosson J, Gaffney EA 2016 A simulation study of sperm motility hydrodynamics near fish eggs and sphere. J. Theor. Biol. 389, 187–197. (10.1016/j.jtbi.2015.10.013) 26542943

[33] Smith DJ, Gaffney EA, Gadêlha H, Kapur N, Kirkman-Brown JC 2009 Bend propagation in the flagella of migrating human sperm, and its modulation by viscosity. Cell Motil. Cytoskeleton 66, 220–236. (10.1002/cm.20345) 19243024

[34] Dresdner RD, Katz DF 1981 Relationships of mammalian sperm motility and morphology to hydrodynamic aspects of cell function. Biol. Reprod. 25, 920–930. (10.1095/biolreprod25.5.920) 7326307

[35] Drobnis EZ, Yudin AI, Cherr GN, Katz DF 1988 Hamster sperm penetration of the zona pellucida: kinematic analysis and mechanical implications. Dev. Biol. 130, 311–323. (10.1016/0012-1606(88)90437-X) 3181633

[36] Ohmuro J, Ishijima S 2006 Hyperactivation is the mode conversion from constant-curvature beating to constant-frequency beating under a constant rate of microtubule sliding. Mol. Reprod. Dev. 73, 1412–1421. (10.1002/mrd.20521) 16894536

[37] Drobnis EZ, Yudin AI, Cherr GN, Katz DF 1988 Kinematics of hamster sperm during penetration of the cumulus cell matrix. Gamete Res. 21, 367–383. (10.1002/mrd.1120210406) 3220430

[38] Klein JD, Clapp AR, Dickinson RB 2003 Direct measurement of interaction forces between a single bacterium and a flat plate. J. Colloid Interface Sci. 261, 379–385. (10.1016/S0021-9797(03)00095-X) 16256545

[39] Suarez SS 2007 Interactions of spermatozoa with the female reproductive tract. Reprod. Fertil. Dev. 19, 103–110. (10.1071/RD06101) 17389139

[40] Ishimoto K, Gaffney EA 2014 Swimming efficiency of spherical squirmers: beyond the Lighthill theory. Phys. Rev. E 90, 012704 (10.1103/PhysRevE.90.012704) 25122332

[41] Ishimoto K, Gaffney EA 2015 Fluid flow and sperm guidance: a simulation study of hydrodynamic sperm rheotaxis. J. R. Soc. Interface 12, 20140172 (10.1098/rsif.2015.0172) PMC4424697 25878133

[42] WHO. 2010 WHO laboratory manual for the examination of human semen and sperm–cervical mucus interaction, 5th edn Geneva, Switzerland: World Health Organization Press.

[43] Katz DF, deMestre NJ 1985 Thrust generation by mammalian spermatozoa against the zona pellucida. Biophys. J. 47, 123a.

[44] Woolley DM 2003 Motility of spermatozoa at surfaces. Reproduction 126, 259–270. (10.1530/rep.0.1260259) 12887282

[45] Miki K, Clapham DE 2013 Rheotaxis guides mammalian sperm. Curr. Biol. 23, 443–452. (10.1016/j.cub.2013.02.007) 23453951 PMC3607503

[46] Bukatin A, Kukhtevich I, Stoop N, Dunkel J, Kantsler V 2015 Bimodal rheotactic behavior reflects flagellar beat asymmetry in human sperm cells. Proc. Natl Acad. Sci. USA 112, 15 904–15 909. (10.1073/pnas.1515159112) PMC4703022 26655343

[47] Babcock DF, Wandernoth PM, Wennemuth G 2014 Episodic rolling and transient attachments create diversity in sperm swimming behavior. BMC Biol. 12, 67 (10.1186/s12915-014-0067-3) 25182562 PMC4354980

[48] Green DPL, Purves RD 1984 Mechanical hypothesis of sperm penetration. Biophys. J. 45, 659–662. (10.1016/S0006-3495(84)84207-1) 6722260 PMC1434915

[49] Coy P, Carcia-Vazquez FA, Visconti PE, Aviles M 2012 Roles of the oviduct in mammalian fertilization. Reproduction 1144, 649–660. (10.1530/REP-12-0279) PMC4022750 23028122

[50] James DF 2009 Boger fluids. Annu. Rev. Fluid Mech. 41, 129–142. (10.1146/annurev.fluid.010908.165125)

[51] Okabe M 2015 Mechanisms of fertilization elucidated by gene-manipulated animals. Asian J. Androl. 17, 646–652. (10.4103/1008-682X.153299) 25851662 PMC4492058

[52] Suarez SS 2016 Mammalian sperm interactions with female reproductive tract. Cell Tissue Res. 363, 185–194. (10.1007/s00441-015-2244-2) 26183721 PMC4703433

[53] Green DPL 1987 Mammalian sperm cannot penetrate the zona pellucida solely by force. Exp. Cell Res. 169, 31–38. (10.1016/0014-4827(87)90221-7) 3817018

