# Supplementary material for: Mechanical tuning of mammalian sperm behaviour by hyperactivation, rheology and substrate adhesion: a numerical exploration
Source: J R Soc Interface. 2016 Nov;13(124):20160633. doi: 10.1098/rsif.2016.0633 (PMC5134015; doi:10.1098/rsif.2016.0633)
Supplement: Supplementary Materials [file rsif20160633supp1.pdf]

Supplementary Material

Mechanical tuning of mammalian sperm behaviour by hyperactivation, rheology and substrate adhesion: a numerical exploration

Kenta Ishimoto[1,2*] and Eamonn A. Gaffney[3]

[1] The Hakubi Center for Advanced Research, Kyoto University, Kyoto, 606-8501, Japan
[2] Research Institute for Mathematical Sciences, Kyoto University, Kyoto, 606-8502, Japan
[3] Wolfson Centre for Mathematical Biology, Mathematical Institute, University of Oxford, Oxford, OX2 6GG, UK

1 Methodological Details

1.1 Further details on the flagellum dimensions and geometry

The virtual spermatozoon is presented in Supplementary Figure 1, consisting of a rigid head, together with a cylindrical flagellum of length $L = 56\mu$m, based on human sperm (e.g. [1]). However, the mammalian sperm flagellum tapers, from about 1 micron in cross sectional diameter in its proximal region (Fig 5 in [2]) to a membrane surrounded axoneme in the distal region. The latter has a diameter slightly larger than, but the same order as that of the axoneme, which is approximately 180nm [3]. However, previous modelling studies have demonstrated that changing the flagellar diameter from 0.1-1.1 microns makes essentially no difference to modelling predictions with the boundary element method and a prescribed waveform (Fig. 4, [4]). Hence we work with a representative flagellar diameter from the distal region of the mammalian sperm; for definiteness, this diameter is taken to be 250nm, giving a virtual flagellum radius of $a = 125$nm.

1.2 The algebraic definition of the flagellar waveforms used in the main text

As in our previous studies [4, 5], we introduce three reference frames to describe the flagellum and its motion: the inertial (laboratory) frame $\{\boldsymbol{\xi}\}$, the body-fixed frame $\{\boldsymbol{\xi}'\}$, and the flagellum frame $\{\boldsymbol{\xi}''\}$ (Supplementary Fig. 1). In addition, we further denote $\mathbf{e}'_i$, $i \in \{1, 2, 3\}$, as the basis vectors associated with the body fixed frame coordinates ξ'_i. In particular, note that the origin of the body fixed frame is at the sperm head-flagellum junction, with $\mathbf{e}'_3$ directed along the positive ξ'_3 axis, which points away from the body fixed frame origin along the sperm head axis of symmetry, as illustrated in Supplementary Fig. 1.

We proceed to consider five types of waveform: non-hyperactivated planar and helical beating, together with hyperactivated beat patterns in low and high viscosity media, distinguishing the beat patterns observed in simple viscoelastic media such as methylcellulose solution [6] and cumulus [7]. The flagellar waveform $\boldsymbol{\xi}'' = (\xi''_1, \xi''_2, \xi''_3)$ is parameterized via

$$\begin{aligned} \xi''_1(\xi''_3, t) &= \alpha \left[A(-\xi''_3)^B + C\right] \cos(k\xi''_3 + \omega t) \\ \xi''_2(\xi''_3, t) &= \left[A(-\xi''_3)^B + C\right] \left[Q + \sin(k\xi''_3 + \omega t)\right], \end{aligned} \quad \text{(S1)}$$

with the coordinate $\xi''_3(s, t)(< 0)$ implicitly determined as a function of arc length $s \in [0, L]$,

$$s(\xi''_3) = \int_{\xi''_3}^{0} \sqrt{1 + \left(\frac{\mathrm{d}\xi''_1}{\mathrm{d}\tilde{\xi}''_3}\right)^2 + \left(\frac{\mathrm{d}\xi''_2}{\mathrm{d}\tilde{\xi}''_3}\right)^2}\, \mathrm{d}\tilde{\xi}''_3. \quad \text{(S2)}$$

*ishimoto@kurims.kyoto-u.ac.jp

Table 1: Reference parameters for the five beat patterns shown in Fig. 1(b). The parameters dictating the non-dimensional scales are given by (i) $\omega_0/[2\pi] = 20$Hz, a typical flagellum frequency [10] (ii) $L = 56\mu$m, a typical human sperm flagellum length [9] and (iii) $\mu_0 = 0.001$Pa·s, essentially the viscosity of water; these are consistent with the definitions in Table 1 of the main text.

Parameter	Interpretation	Beat I	Beat II	Beat III	Beat IV	Beat V
Beat type	Flagellar waveform	Planar (non-hyper-activated)	Helical (non-hyper-activated)	Hyper-activated (low viscosity)	Hyper-activated (high viscosity)	Hyper-activated (in cumulus)
α	beat chirality parameter	0	0.2 [9]	0	0	0
A	beat amplitude	0.2 [9]	0.2 [9]	0.4 [13]	0.15 (this study)	-0.15 (this study)
B	envelope parameter	1	1	1	4 (this study)	1
C/L	envelope parameter	0	0	0	0	0.1(this study)
Q	beat asymmetry parameter	0	0	1	0	1
kL	wavenumber	3π [9]	3π[9]	1.43π [13]	8π (this study)	2π (this study)
ω/ω_0	beat frequency	1[1]	1 [1]	0.5 [10]	0.2 [1]	0.3 [12]
μ/μ_0	viscosity	1	1	1	4000 [6]	100 [12]
	PRIMARY SOURCES	[6, 8]	[6, 8]	[11]	[6]	[7]

The parameter sets associated with the flagellar waveforms are summarized in Supplementary Table 1 and snapshots of these flagellar shapes are shown in Fig. 2(b) of the main text, taken from the numerical simulation described below.

In particular, for beat forms I and II which are non-hyperactivated planar and helical beats respectively, the primary reference sources are Dresdner *et al.* [8] and Suarez *et al.* [6], with further derived parameters chosen to ensure a matching with observed waveforms given by Smith *et al.* [9]. Analogous remarks apply for beat patterns III, IV, V, which respectively correspond to hyperactivation in a low viscosity medium, a simple high viscosity medium such as methylcellulose solution and the matrix of the Cumulus-Oophorus-Cell, or cumulus for short. The primary data for the waveforms is taken from several studies [6, 7, 10, 11, 12], together with derived parameters from Curtis *et al.* [13] and further derived parameters in this study to ensure the virtual waveforms closely match the observed ones.

In addition, the mapping between the flagellar frame and the body fixed frame is required below; it is determined by the constraint that there is no flagellar bending at the head-flagellar junction, as observed in mammalian sperm (e.g. [1]). Hence the constraint can be written as

$$\boldsymbol{\xi}''|_{s=0} = \boldsymbol{\xi}'|_{s=0}, \qquad \mathrm{d}\boldsymbol{\xi}''/\mathrm{d}s|_{s=0} = -\mathbf{e}'_3, \tag{S3}$$

where $\mathbf{e}'_3$ is the unit body fixed basis vector directed along the positive ξ'_3 axis, pointing away from the head-flagellum junction along the sperm head axis of symmetry and $\boldsymbol{\xi}'$, $\boldsymbol{\xi}''$ are the flagellum centreline locations relative to the body fixed and flagellar frames respectively.

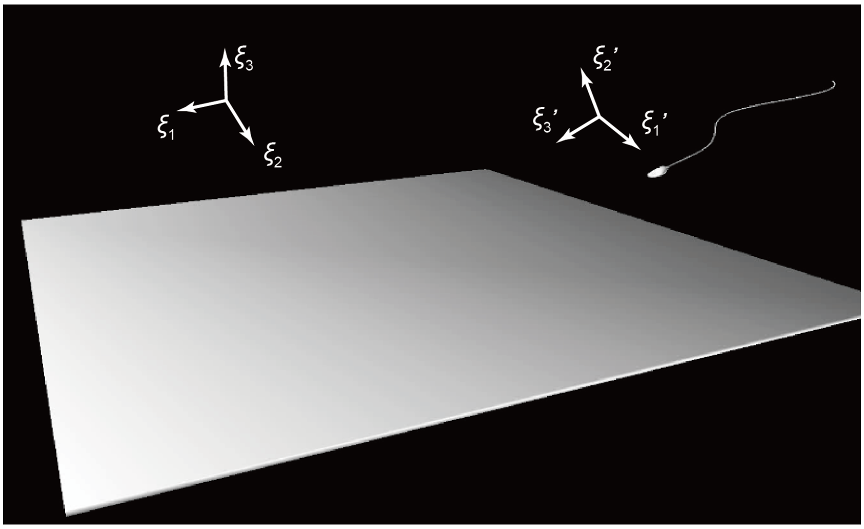

Figure 1: A snapshot of the numerical simulation for a swimming spermatozoon in the vicinity of a rigid wall, with the laboratory reference frame $\{\boldsymbol{\xi}\}$ and the body-fixed frame $\{\boldsymbol{\xi}'\}$ shown.

1.3 The numerical solution of the equations for the motion of the sperm.

1.3.1 Mesh generation.

The model human sperm head is a deformed ellipsoid of dimension 1.1μm $\times$ 2.8μm $\times$ 4.5μm, as used in our previous studies [4, 5]. Its mesh is generated by applying the mapping detailed in Ishimoto and Gaffney [4] to an ellipsoid generated by stretching a meshing of a sphere. In turn, the latter is formed by $N_{div} = 2$ divisions of a spherical surface in the BEMLIB library meshing subroutines accompanying Pozrikidis' text [14].

The flagellum is interpolated by a hexagonal tube with hemispherical caps, as implemented in our previous studies [4, 5]. The centreline of the flagellum is discretized and each position is provided in the flagellar frame via the waveform given by equations (S1) and (S2), and the circular cross section of the flagellum is generated by the normal and the binormal vectors for a given point at the centreline. In addition, the flagellum is mapped into the body fixed frame so that the head and the flagellum are connected with the axis of symmetry of the head parallel to the tangent of the proximal flagellum, as summarised by equations (S3). However, in practice, the flagellum is given a separation from the sperm head of $0.005L$ in the tangential direction for numerical convenience; this completes the definition of the cell mesh in its body-fixed reference frame and there is a total of $N = 494$ mesh points for the whole cell.

1.3.2 Numerical solution via the boundary element method.

We assume the sperm cell is swimming in a Newtonian fluid, and thus the low Reynolds number flow around the spermatozoon is governed by the Stokes equations, which are numerically solved via the boundary element method. With the assumption that the cell does not change its volume, which is implicit in the above meshing, the flow velocity can be obtained by the single-layer boundary integral, via [15]

$$u_i(\boldsymbol{x}) = -\frac{1}{8\pi\mu}\int_S G_{ji}(\boldsymbol{x}, \boldsymbol{x}')q_j(\boldsymbol{x}')\,\mathrm{d}S_{\boldsymbol{x}'}, \tag{S4}$$

for $i, j \in \{1, 2, 3\}$, with S denoting the cell surface, including both the head and the flagellum. The unknown vector $\boldsymbol{q}$ denotes the difference of the internal and external traction, $\boldsymbol{q} = \boldsymbol{f}_{hyd} - \boldsymbol{f}_{int}$, and μ is the viscosity. The

integral kernel, G_{ij} is the Stokeslet in the absence of external domain boundaries, and the Blakelet [16] in the presence of an infinite plane wall. The kernel for a flow external to a no-slip sphere is also known and is given explicitly by Higdon [17].

The fluid velocity on the left-hand side of the boundary integral relation, $\boldsymbol{u}(\mathbf{x})$ is the same as the cell surface velocity, by the no-slip condition, and is relative to the inertial (laboratory) frame. It can be constructed from the velocity, $\boldsymbol{U}$, and the angular velocity, $\boldsymbol{\Omega}$, relating the inertial frame and the body-fixed frame, together with the deformation velocity of the flagellum, $\tilde{\boldsymbol{u}}$. The latter, relative to the flagellar frame, can be obtained from the waveform given by equations (S1) and (S2), but one must map this velocity to the inertial frame. The location and orientation of the body fixed frame relative to the inertial frame is known from the initial conditions at the first timestep and otherwise from the results of the previous timestep. Thus, finally, one requires the relation between the flagellar frame and the body fixed frame, which can be constructed from the above-mentioned head-flagellum junction conditions. Recall these are

$$\boldsymbol{\xi}''|_{s=0} = \boldsymbol{\xi}'|_{s=0}, \qquad \mathrm{d}\boldsymbol{\xi}''/\mathrm{d}s|_{s=0} = -\boldsymbol{e}_3',$$

where s is flagellum arclength, with $s = 0$ corresponding to the head-flagellum junction and $\boldsymbol{\xi}'$, $\boldsymbol{\xi}''$ are the flagellum centreline locations relative to the body fixed and flagellar frames respectively, while $\boldsymbol{e}_3'$ is a unit body fixed basis vector directed along the positive ξ_3' axis. Hence one can construct $\boldsymbol{u}(\mathbf{x})$ at any point on the cell surface, and thus equation (S4) constitutes an integral equation for $\boldsymbol{q}$.

These boundary integral equations are also supplemented with the force and torque balance equations

$$\boldsymbol{F} = \int_S [\boldsymbol{q}(\boldsymbol{x}') + \boldsymbol{f}_{wall}(\boldsymbol{x}') + \boldsymbol{f}_{ad}(\boldsymbol{x}')] \, \mathrm{d}S_{\boldsymbol{x}'} = \mathbf{0} \tag{S5}$$

$$\boldsymbol{T} = \int_S (\boldsymbol{x'} - \boldsymbol{X}_0) \times [\boldsymbol{q}(\boldsymbol{x}') + \boldsymbol{f}_{wall}(\boldsymbol{x}') + \boldsymbol{f}_{ad}(\boldsymbol{x}')] \, \mathrm{d}S_{\boldsymbol{x}'} = \mathbf{0}, \tag{S6}$$

where $\boldsymbol{X}_0$ is the location of the head-flagellum junction. While the torque is therefore not relative to the origin of an inertial frame, the absence of a net force on the swimmer due to the absence of inertia entails one can resolve torques about any point without loss of generality.

Thus (S4), (S5) and (S6) form a closed linear system of $3N + 6$ scalar equations, where N is the number of mesh points for the discretization of the cell surface. The $3N + 6$ scalar unknowns are the linear and angular velocities, $\boldsymbol{U}$ and $\boldsymbol{\Omega}$ together with the vector surface tractions $\boldsymbol{q}(\boldsymbol{x})$ at the N mesh points, all of which can thus be numerically obtained at each time step using an LU decomposition. Noting that the body fixed frame is defined such that head-flagellum junction is at its origin and the sperm head is fixed in this frame, one can thus time march the location of head flagellum junction and orientation of the sperm head at each-step and thus also time march the body fixed frame. In turn, this provides enough information to reconstruct the flagellar location in the inertial frame at each timestep. This time-marching is implemented via the Heun time-stepping method, with timestep $\Delta t/T = 1/800$, where $T = 2\pi/\omega$ is the beat period. Further details on the simulation computation can also be found in previous work [15], with additional model validation presented in the supplementary material of Ishimoto and Gaffney [18].

Note that for a sperm clamped to a substrate, such that it is fixed at a point on the substrate and does not rotate relative to this point, which represents adherence with a sufficiently large binding strength, the boundary element algorithm is marginally different. In particular, with the origin of the body fixed frame redefined to be the point of contact and a suitable redefinition of the body fixed frame orientation, the linear and angular velocity between the laboratory and the body-fixed frames, $\boldsymbol{U}$, and $\boldsymbol{\Omega}$, are both zero. Hence, the N traction vectors $\boldsymbol{q}$ can be determined by the single layer equation (S4). Thus, in turn, the hydrodynamic traction, $\boldsymbol{f}_{hyd}$, for such a cell can also be determined, without recourse to the global linear and angular momentum balance for the whole cell.

1.3.3 Computation of the mechanical power output

We define the mechanical power output of the cell by $P = \int_S \boldsymbol{f}_{hyd}(\boldsymbol{x}') \cdot \boldsymbol{u}(\boldsymbol{x}')\, \mathrm{d}S_{\boldsymbol{x}'}$, where the hydrodynamic traction is obtained from the traction difference via [19]

$$f_{hyd,i}(\boldsymbol{x}) = -\frac{1}{2} q_i(\boldsymbol{x}) + \frac{1}{8\pi} n_k(\boldsymbol{x})\, \mathrm{pv} \int_S T_{ijk}(\boldsymbol{x}, \boldsymbol{x}') q_j(\boldsymbol{x})\, \mathrm{d}S_{\boldsymbol{x}'}.$$

Here, $\boldsymbol{n}$ is the unit outward normal of the cell surface and "pv" denotes the principal value integral. The rank-three tensor T_{ijk} is the stress Green function, given by $T_{ijk}(\boldsymbol{x}, \boldsymbol{x}') = -6 r_i r_j r_k / |\boldsymbol{r}|^5$ with $r_i = x_i - x_i'$ for a cell swimming in a free space, with known analogous expressions in the presence of a wall or a sphere [19]. The principal value integral in the computation of the power output is determined with contributions from the mesh element containing the singularity defined to be zero, yielding an approximation that is asymptotically accurate in the reciprocal of the number of mesh elements [15] and the computational results are shown in Fig. 3 of the main text.

1.4 Force calculation for a clamped sperm beating in a linear viscoelastic medium

The linear viscoelastic Maxwell model, which is a good representation of methylcellulose solution rheology [1], is characterised by the constitutive relation

$$\mathcal{L}\boldsymbol{\tau} := \lambda \frac{\partial \boldsymbol{\tau}}{\partial t} + \boldsymbol{\tau} = \mu \dot{\boldsymbol{\gamma}},$$

where $\boldsymbol{\tau}$ is the deviatoric stress, $\dot{\boldsymbol{\gamma}}$ is the rate of strain, μ is the viscosity and λ is the elastic relaxation time. Note also that this reduces to the Newtonian constitutive relation as the relaxation time λ tends to zero and that $\boldsymbol{\tau}^{Stokes} = \mathcal{L}\boldsymbol{\tau}$ where $\boldsymbol{\tau}^{Stokes}$ is the deviatoric stress for Newtonian flow.

Linear momentum balance yields $\nabla \cdot \boldsymbol{\sigma} = 0$, where

$$\boldsymbol{\sigma} = -p\mathbf{1} + \boldsymbol{\tau}$$

is the stress tensor so that one has $\nabla \mathcal{L} p = \mu \Delta \boldsymbol{u}$, together with the incompressibility constraint $\nabla \cdot \boldsymbol{u} = 0$. Combined with no-slip boundary for $\boldsymbol{u}$ on the surface of a microswimmer and decay of the velocity field at spatial infinity, one has the solution for the viscoelastic flow associated with a clamped microswimmer in terms of the analogous Stokes flow solution is given by

$$\boldsymbol{u} = \boldsymbol{u}^{Stokes}, \qquad p = \mathcal{L}^{-1} p^{Stokes}, \tag{S7}$$

where the superscript 'Stokes' denotes the Newtonian, Stokes equation, solution. One should note that equation (S7) does not hold for a free swimmer subject to external forces as the non-Newtonian constitutive relation enters into the form of the total force and moment balance for the cell and thus the viscoelastic and Newtonian predictions for the velocity and angular velocity of the cell motion differ. Hence we only proceed to consider the forces imposed on the substrate by a clamped sperm with its flagellum beating in a viscoelastic medium, with a non-trivial elastic relaxation time.

The region external to the cell is defined by $\Omega(t)$ and its boundary $\partial\Omega(t)$ is given by the union of S, the surface of the cell, together with $\partial\Omega_*$, which is the time-independent boundary consisting of the wall and the far boundary at spatial infinity. The force exerted by a spermatozoon clamped to a wall and beating in a linear viscoelastic Maxwell fluid is given by

$$\boldsymbol{F}_{VE} = \int_S \boldsymbol{\sigma} \cdot \boldsymbol{n}\, dS = \int_S \mathcal{L}^{-1}(-p^{Stokes}\mathbf{1} + \boldsymbol{\tau}^{Stokes}) \cdot \boldsymbol{n} dS,$$

where $\boldsymbol{n}$ is the outward unit normal to the surface. Using the equations of motion and Gauss' integral theorem, the time derivative of the force is given by

$$\begin{aligned}\frac{\partial}{\partial t}\boldsymbol{F}_{VE} &= \frac{\partial}{\partial t}\left(\int_{\partial\Omega_*}\boldsymbol{\sigma}\cdot\boldsymbol{n}\,dS - \int_{\Omega(t)}\nabla\cdot\boldsymbol{\sigma}\,dS\right) = \int_{\partial\Omega_*}\frac{\partial\boldsymbol{\sigma}}{\partial t}\cdot\boldsymbol{n}\,dS \\ &= \int_S \frac{\partial\boldsymbol{\sigma}}{\partial t}\cdot\boldsymbol{n}\,dS + \int_{\Omega(t)}\nabla\cdot\frac{\partial\boldsymbol{\sigma}}{\partial t}\,dV = \int_S\frac{\partial\boldsymbol{\sigma}}{\partial t}\cdot\boldsymbol{n}\,dS.\end{aligned}$$

We therefore have the relations,

$$\mathcal{L}\boldsymbol{F}_{VE} = \int_S \mathcal{L}\boldsymbol{\sigma}\cdot\boldsymbol{n}\,dS = \int_S \mathcal{L}\mathcal{L}^{-1}\left(-p^{Stokes}\mathbf{1} + \boldsymbol{\tau}^{Stokes}\right)\cdot\boldsymbol{n}\,dS = \boldsymbol{F}_{Stokes}, \tag{S8}$$

where $\boldsymbol{F}_{Stokes}$ corresponds to the force exerted on the wall by the spermatozoon if it was clamped to a wall and beating in a Newtonian fluid, and thus can be determined from the boundary element simulations. Hence, integrating equation (S8) with respect to time gives the force exerted by a spermatozoon clamped to a wall and beating in a linear viscoelastic Maxwell fluid via

$$\boldsymbol{F}_{VE} = \boldsymbol{F}_{Stokes}(0)e^{-t/\lambda} + \int_0^t \frac{1}{\lambda}e^{-(t-t')/\lambda}\boldsymbol{F}_{Stokes}(t')\,dt',$$

which completes the derivation of equation (2.5) in the main text.

2 Model robustness

In this section we briefly consider whether the modelling conclusions are specific to fine tuning many aspects of the virtual sperm model, including the head geometry, the flagellar length, the beat pattern frequency and also the binding parameters.

2.1 Scalings

Firstly, note that the non-dimensional model represents a three-parameter family of dimensional models, and hence parameter changes can be often incorporated into changes between the scalings relating the dimensional and non-dimensional models.

Thus, consider a different sperm with an order one change in its flagellar length, for instance hamster sperm are significantly longer than human sperm [20]. This simply changes the lengthscale and forcescale used to map between the dimensional and non-dimensional model: predicted trajectories are unchanged. With the flagellum of unit length in the non-dimensional model, one thus has an order one change in the size of the sperm head. However, the modelling demonstrates a lack of qualitative sensitivity to sperm head, as indicated below (and also by comparison with the point-head sperm of Simons et. al. [21]). Furthermore, force ratios comparing sperm before and after hyperactivation or the presence of cumulus, are unchanged as only scalings have been altered.

Hence, since the conclusions are all qualitative and comparative in that they do not rely on quantitative details but on trends and comparisons between virtual sperm in different scenarios, this highlights that there is a lack of sensitivity in the modelling conclusions for changes in the lengthscale of the sperm. Analogous remarks hold for the timescale and forcescale of the virtual sperm and thus many of the parameters in Table 1 of the Supplementary Material. In the next section, we proceed to consider model sensitivity to changes in the sperm head and binding parameters which are not readily incorporated into the dimensional scalings.

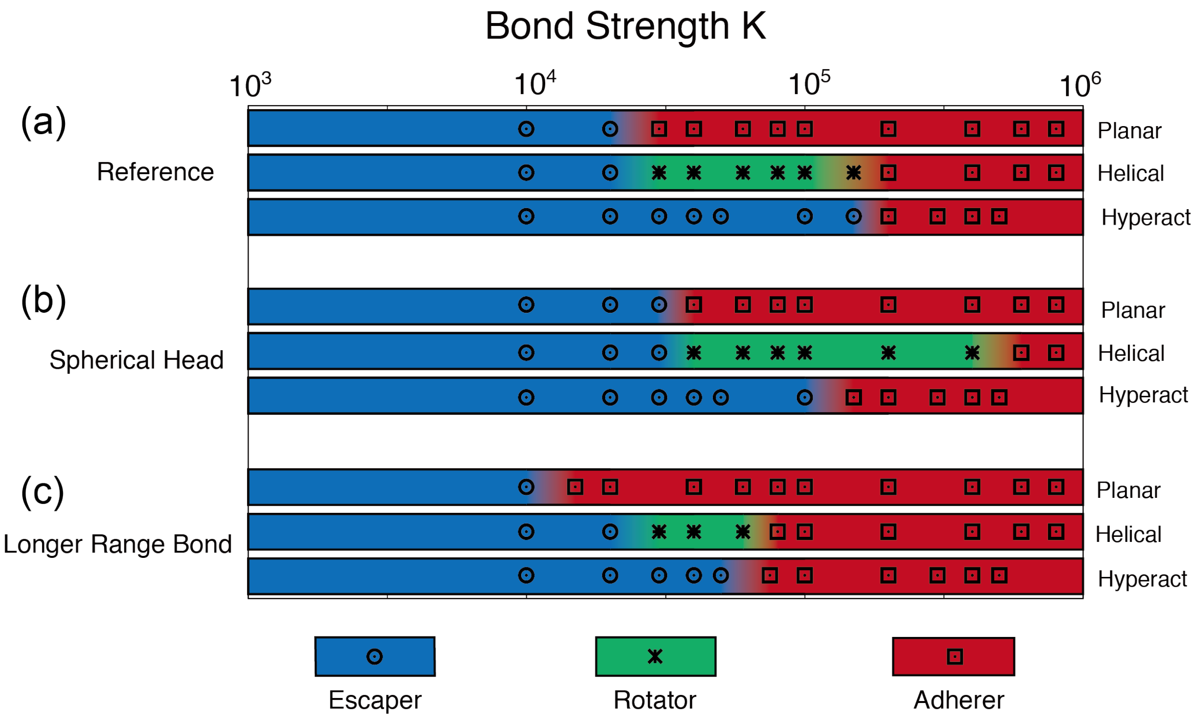

Figure 2: The long-time behaviour of sperm is presented for the beat patterns I (planar), II (helical) and III (low-viscosity hyperactivated) given a spectrum of bond strengths, with the regions between the different final sperm behaviours highlighted by a transition in colour. These transitions of colour indicate that the long time behaviour changes somewhere within this region, noting that only a discrete number of bond strengths have been simulated. (a) This gives the long time behaviour for the virtual sperm simulated with parameters from Table 1 of the main text and waveform parameters from Supplementary Table 1 above. It is the same as plot 5(d) in the main text and reproduced here for ease of reference. (b) The head geometry of the virtual sperm is changed to that of a sphere with the same volume as the representative human sperm head used in the main text simulations. (c) The virtual sperm is that of the main text but the lengthscale of bond release is almost doubled to $R_{off} = 0.021L$ with all other parameters unchanged. Note that the qualitative features of the long time behaviour are not changed by these alterations in sperm head geometry or bond release length, though the reduced contact area with a spherical head entails transitions occur for stronger bonds while, in contrast, the larger lengthscale of bond release entails transitions at weaker bond strengths.

2.2 Long time behaviour for sperm in the presence of longer range adhesive bonds or with a spherical head

Supplementary Fig. 2 presents the long time behaviour for sperm encountering an adhesive substrate for two different sperm head geometries and a range of binding parameter values. In particular, plot(a) reproduces Fig. 5(d) of the main text for ease of reference, which corresponds to simulations with the planar, helical and the low-viscosity hyperactivated beat patterns, corresponding to waveforms I, II, III of main text Fig. 2(b) using the scales from Table 1 of the main text and the waveform parameters of Supplementary Table 1 above.

In contrast, plot (b) of Supplementary Fig. 2 has the same parameter values and waveforms, but the sperm head is now a sphere with the same volume of the representative human sperm head used in the main text; thus the head radius is approximately 1.21μm. The final sperm behaviour is still qualitatively the same, despite the change in geometry, though the transitions between different surface behaviours occur at larger bond strengths due to a decrease in the head-substrate contact area with a spherical geometry, as the head orients to present its flattened side to the substrate when the representative human sperm head geometry of the main text is used.

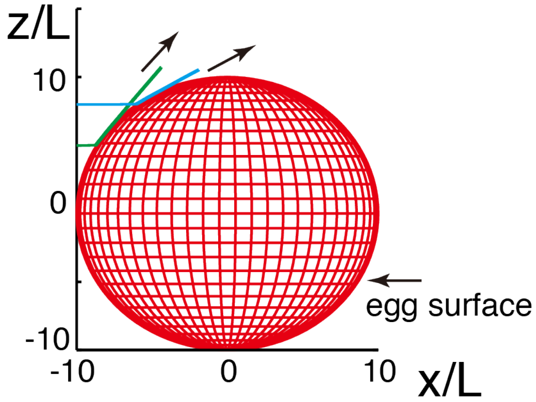

Figure 3: Predicted sperm trajectories in the presence of a weakly adhesive sphere of radius $R = 10L$, which is representative of a human egg radius. The trajectories start of the left and the sperm possesses the non-hyperactivated planar beat pattern depicted by waveform I of Fig. 2(b) in the main text, with the bond strength given by $K = 10^3$. All other scales are given by Table 1 of the main text, with planar waveform parameters for beat pattern I given in Supplementary Table 1 above

This increase in bond strength before transition is particularly noticeable for switching between surface cycling and adherence for the helical beater, as the bonds tend to be more stretched and thus easier to break during the spherical head movement on the substrate, in comparison with a flattened head, oriented flat side down.

Supplementary Fig. 2(c) shows the results for the virtual sperm used in the main text but now with an increase in the adhesion bond breaking lengthscale, which is now given by $R_{off} = 0.021L$ and is nearly twice as large as the value used in the main text; all other parameters are unchanged. Once more, the long time sperm behaviour is qualitatively the same, though transitions between different behaviours occur at lower bond strengths due to the increased range of the bonding.

More generally, these results emphasise that the qualitative features of predicted sperm behaviour near an adhesive substrate are not sensitive to fine scale details of head geometry or bond parameters, though these do impact on the local interaction of the sperm and substrate and thus alter the quantitative predictions for thresholds that induce changes in the virtual sperm behaviour.

3 Swimming trajectories in the presence of an adhesive sphere

In Supplementary Fig. 3 two trajectories are determined for a sperm swimming into a weakly adhesive sphere ($K = 10^3$) for the planar beat pattern, waveform I of Fig. 2(b) in the main text, and starting from the locations $(x, y, z) = (-10L, 0, 5L)$ and $(-10L, 0, 8L)$ with the sperm head directed along the positive x axis. The sphere is centred at the origin and has radius $10L$, where $L = 56\mu$m is the flagellum length so that $10L$ is a typical human egg radius. All other scales are given by Table 1 of the main text, with planar waveform parameters, beat pattern I, from Supplementary Table 1 above. For both cases, the sperm escapes from the sphere, and thus is effectively scattered, though the local dynamics is only negligibly dependent on the sphere surface curvature and this has been observed more generally, justifying the neglect of egg surface curvature when considering sperm adhesive dynamics near substrates representing the zona pellucida in the main text. Nonetheless, when the binding is sufficiently weak that the cell does not adhere to the sphere, the subsequent cell behaviour with swimming in the proximity of the surface is associated with a weaker cell-surface interaction due to the surface curvature, which significantly reduces the duration the sperm swims close to the egg compared to flat substrate with the same adhesion binding strength.

4 Movie captions

Supplementary Movie 1:
Simulation movie of sperm swimming for the non-hyperactivated planar waveform, beat pattern I of Fig 2(b) in the main text. The bond strength is $K = 10^5$, and stable cell adhesion is achieved.

Supplementary Movie 2:
Simulation movie of sperm swimming for the non-hyperactivated helical waveform, beat pattern II of Fig 2(b) in the main text. The bond strength is $K = 10^5$, and the cycling motion across the substrate while the sperm is still bound can be observed.

Supplementary Movie 3:
Simulation movie of sperm swimming with beat pattern III of Fig 2(b) in the main text, a hyperactivated asymmetric waveform. The bond strength is $K = 5 \times 10^4$, and the cell finally detaches from the wall.

Supplementary Movie 4:
Simulation movie of the hyperactivated swimming in a high viscosity medium with beat pattern IV of main text Fig. 2(b). The bond strength is $K = 1 \times 10^7$, and the cell stably adheres to the wall.

Supplementary Movie 5:
Simulation movie of hyperactivated swimming within a highly viscous medium for beat pattern V of main text Fig 2(b), which is observed in cumulus. The bond strength is $K = 3 \times 10^7$, and the cell stably adheres to the wall.

References

[1] D. J. Smith, E. A. Gaffney, H. Gadêlha, N. Kapur, and J.C. Kirkman-Brown. Bend propagation in the flagella of migrating human sperm, and its modulation by viscosity. *Cell Motility and the Cytoskeleton*, 66:220–236, 2009.

[2] D. M. Woolley. Motility of spermatozoa at surfaces. *Reproduction*, 126:259–270, 2003.

[3] C. B. Lindemann and D. R. Mitchell. Evidence for axonemal distortion during the flagellar beat of chlamydomonas. *Cell Motility and Cytoskeleton*, 64:580–589, 2007.

[4] K. Ishimoto and E. A. Gaffney. A study of spermatozoan swimming stability near a surface. *Journal of Theoretical Biology*, 360:187–199, 2014.

[5] K. Ishimoto, J. Cosson, and E. A. Gaffney. A simulation study of sperm motility hydrodynamics near fish eggs and sphere. *Journal of Theoretical Biology*, 389:187–197, 2016.

[6] S. S. Suarez and X. Dai. Hyperactivation enhances mouse sperm capacity for penetrating viscoelastic media. *Biology of Reproduction*, 46(4):686, 1992.

[7] E. Z. Drobnis, A. I. Yudin, G. N. Cherr, and D. F. Katz. Hamster sperm penetration of the zona pellucida kinematic analysis and mechanical implications. *Developmental Biology*, 130(1):311–323, 1988.

[8] R. D. Dresdner and D. F. Katz. Relationships of mammalian sperm motility and morphology to hydrodynamic aspects of cell function. *Biology of Reproduction*, 25:920–930, 1981.

[9] D. J. Smith, E. A. Gaffney, J. R. Blake, and J. C. Kirkman-Brown. Human sperm accumulation near surfaces: a simulation study. *Journal of Fluid Mechanics*, 621:289–320, 2009.

[10] E. H. Ooi, D. J. Smith, H. Gadêlha, E. A. Gaffney, and J. Kirkman-Brown. The mechanics of hyperactivation in adhered human sperm. *Royal Society Open Science*, 1:140230, 2014.

[11] J. Ohmuro and S. Ishijima. Hyperactivation is the mode conversion from constant-curvature beating to constant-frequency beating under a constant rate of microtubule sliding. *Molecular Reproduction and Development*, 73:1412–1421, 2006.

[12] E. Z. Drobnis, A. I. Yudin, G. N. Cherr, and D. F. Katz. Kinematics of hamster sperm during penetration of the cumulus cell matrix. *Gamete Research*, 21:367–383, 1988.

[13] M. P. Curtis, J. C. Kirkman-Brown, T.J. Connolly, and E.A. Gaffney. Modelling a tethered mammalian sperm cell undergoing hyperactivation. *Journal of Theoretical Biology*, 309:1–10, 2012.

[14] C. Pozrikidis. *A Practical Guide to Boundary Element Methods with the Software Library BEMLIB*. CRC, 2002.

[15] K. Ishimoto and E. A. Gaffney. Swimming efficiency of spherical squirmers: Beyond the Lighthill theory. *Physical Review E*, 90:012704, 2014.

[16] J. R. Blake. A note on the image system for a stokeslet in a no slip boundary. *Proceedings of the Cambridge Philosophical Society*, 70:303–310, 1971.

[17] J. J. L. Higdon. A hydrodynamic analysis of flagellar propulsion. *Journal of Fluid Mechanics*, 90:685–711, 1979.

[18] K. Ishimoto and E. A. Gaffney. Fluid flow and sperm guidance: a simulation study of hydrodynamic sperm rheotaxis. *Journal of the Royal Society Interface*, 12:20140172, 2015.

[19] C. Pozrikidis. *Boundary Integral and Singularity Methods for Linearized Viscous Flow*. Cambridge University Press, 1992.

[20] J. M. Cummins. Sperm size, body mass and reproduction in mammals. In J. Andre, editor, *The sperm cell: fertilizing power, surface properties, motility, nucleus acrosome evolutionary apsects*, pages 395–398. Martinus Nijhoff Publishers, 1982.

[21] J. Simons, S. Olson, R. Cortez, and L. Fauci. The dynamics of sperm detachment from epithelium in a coupled fluid-biochemical model of hyperactivated motility. *Journal of Theoretical Biology*, 354:81–94, 2014.
